# Haplotypes with Copy Number and Single Nucleotide Polymorphisms in *CYP2A6* Locus Are Associated with Smoking Quantity in a Japanese Population

**DOI:** 10.1371/journal.pone.0044507

**Published:** 2012-09-25

**Authors:** Natsuhiko Kumasaka, Masayuki Aoki, Yukinori Okada, Atsushi Takahashi, Kouichi Ozaki, Taisei Mushiroda, Tomomitsu Hirota, Mayumi Tamari, Toshihiro Tanaka, Yusuke Nakamura, Naoyuki Kamatani, Michiaki Kubo

**Affiliations:** 1 Research Group for Medical Informatics, Center for Genomic Medicine, RIKEN, Tokyo, Japan; 2 Research Group for Genotyping, Center for Genomic Medicine, RIKEN, Yokohama, Kanagawa, Japan; 3 Research Group for Disease-causing Mechanism, Center for Genomic Medicine, RIKEN, Yokohama, Kanagawa, Japan; 4 Research Group for Pharmacogenomics, Center for Genomic Medicine, RIKEN, Yokohama, Kanagawa, Japan; 5 Human Genome Center, Institute of Medical Science, The University of Tokyo, Tokyo, Japan; IPO -, Inst Port Oncology, Portugal

## Abstract

Smoking is a major public health problem, but the genetic factors associated with smoking behaviors are not fully elucidated. Here, we have conducted an integrated genome-wide association study to identify common copy number polymorphisms (CNPs) and single nucleotide polymorphisms (SNPs) associated with the number of cigarettes smoked per day (CPD) in Japanese smokers (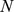 = 17,158). Our analysis identified a common CNP with a strong effect on CPD (rs8102683; 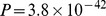) in the 19q13 region, encompassing the *CYP2A6* locus. After adjustment for the associated CNP, we found an additional associated SNP (rs11878604; 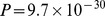) located 30 kb downstream of the CYP2A6 gene. Imputation of the *CYP2A6* locus revealed that haplotypes underlying the CNP and the SNP corresponded to classical, functional alleles of CYP2A6 gene that regulate nicotine metabolism and explained 2% of the phenotypic variance of CPD (ANOVA 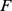-test 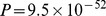). These haplotypes were also associated with smoking-related diseases, including lung cancer, chronic obstructive pulmonary disease and arteriosclerosis obliterans.

## Introduction

Smoking is a common risk factor for many diseases and a leading cause of mortality [1]. It is well known that smoking persistence, smoking quantity and nicotine dependence are highly heritable traits, and approximately 30–80% of inter-individual variance is attributable to genetic factors [2], [3]. Recently, genome-wide association studies (GWAS) and genome-wide meta-analyses have identified several genetic loci that are associated with smoking quantity (as estimated by the number of cigarettes smoked per day, CPD), smoking initiation, smoking cessation and age of smoking initiation [4]–[6]. However, these studies were conducted in subjects of European descent, and few GWAS have been performed in any Asian population, even though this group accounts for two-thirds of the world population. Thus, studies in Asian populations might provide novel insight into the genetic architecture of smoking behavior and smoking-related diseases. Here, we report a large-scale GWAS and a replication study examining CPD in 17,158 Japanese subjects. We assessed genome-wide single-nucleotide polymorphisms (SNPs) along with common copy number polymorphisms (CNPs) and identified haplotypes with a SNP and a CNP at the *CYP2A6* locus that is a strong susceptibility variant for CPD and smoking-related diseases. Our study also estimated the heritability explained by the haplotype for CPD and smoking-related disease traits.

## Results

We enrolled 11,696 Japanese subjects in the GWAS for CPD (Table S1) with the support of the BioBank Japan Project [7]. Stringent quality control criteria for both CNPs and SNPs, including principal component analysis (PCA), were applied as described previously [8], [9]. To extend the genomic coverage, genome-wide imputation was performed for SNPs using data from HapMap samples (JPT + CHB; Phase II). Consequently, the genotype data for 4,256 autosomal CNPs and 2,312,503 autosomal SNPs with minor allele frequencies (MAF) 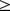
0.01 were obtained (see Materials and Methods for details). Each CNP or SNP was then evaluated for association with CPD using a linear regression model that accounted for the additive effects of copy number dosage or allele dosage on CPD with other covariates. Although no significant population stratification was suggested by the data from our study population (Figure S1), we also used the first two eigenvectors within the East Asian population (Figure S2) as covariates. The Quantile-Quantile plot of the 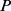-values exhibited an inflation factor (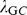; [10]) of 1.01 for the genome-wide SNPs (Figure S3a), which suggests that there was no additional population stratification in our population. In addition, the Quantile-Quantile plot for the genome-wide CNPs also exhibited an inflation factor of 1.05 (Figure S3b), which suggests that there is minimal genotyping error [11] in the CNP data.

**Figure 1:**
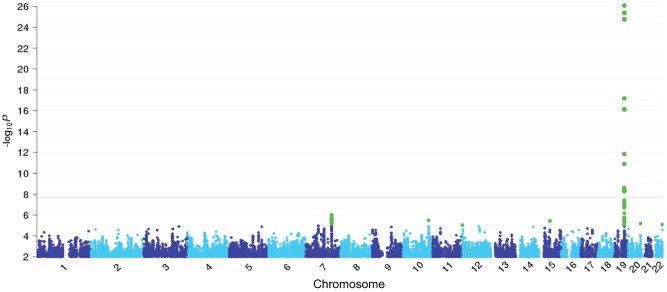
Manhattan plot showing the significance of association for all CNPs and SNPs in the genome-wide CPD analysis. The SNPs consist of both Illumina 610K chip and imputed HapMap SNPs. All CNPs and SNPs are plotted on the 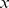 axis according to their positions on each chromosome against association with CPD on the 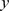 axis (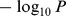-value). SNPs and CNPs with 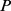-values 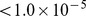 are highlighted in green. A dark gray line shows the genome-wide significance level after Bonferroni's correction with a total of 2,312,503 SNPs and 4,256 CNPs.

Our GWAS identified a significant association on 19q13 that satisfied the genome-wide significance threshold of 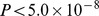 (Figure 1 and Table S2). This region encompasses a series of CYP2 family genes (Figure S4) and one of the most significantly associated markers was a CNP (rs8102683; 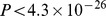; Table S2), which is located 10 kb upstream of the CYP2A6 gene. Four additional CNPs were also clustered at this locus, and these five CNPs were in strong linkage disequilibrium (LD) with each other (Figure S5). Moreover, haplotype estimation revealed that the five CNPs shared a common deletion (frequency 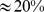; Table S3). These findings suggest that the five CNPs are located within the same copy number variation region. In fact, the depth of coverage for the 89 Japanese subjects from the 1000 Genomes Project (Phase I 2011-11-23; see URLs) clearly showed the common deletion region ranging from the 3′ end of the CYP2A6 gene to the 3′ end of the CYP2A7 gene (Figure S6), which is a region that encompasses all five CNP markers. Since the CYP2A6 gene encodes a nicotine-metabolizing enzyme [12], [13], it is reasonable to speculate that this common deletion may directly cause a loss of function of the CYP2A6 gene that would result in slow nicotine metabolism. Indeed, the estimated effect size of one copy of the CYP2A6 gene corresponding to approximately three cigarettes per day (Table S2 and Figure S7).

For *in silico* replication, we selected the *CYP2A6* locus, along with five genome-wide loci for CPD that had 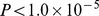, in an additional independent sample of 5,462 Japanese subjects from the BioBank Japan Project (Table S1). We assessed the CPD association with the CNP (rs8102683) at the *CYP2A6* locus and five representative SNPs for the other loci; only the CNP (rs8102683) at the *CYP2A6* locus was significantly replicated (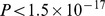; Table S2). A combined analysis of the GWAS and the replication study showed a strong association at the *CYP2A6* locus (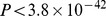), suggesting that this locus is the only major quantitative trait locus associated with CPD in the Japanese population.

It is known that the genotyping error rate of CNPs is higher than that of SNPs [8]. To validate the CNP genotyping quality, we performed TaqMan gene copy number assays using real-time quantitative PCR (referred to as “TaqMan assays” throughout this manuscript) for the five CNPs at the *CYP2A6* locus. Using subsamples from both the GWAS (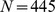) and the replication set (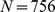), we found a concordance rate of more than 95% for all CNPs (Table S4). In particular, the CNP (rs8102683) showed the highest concordance rate (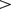
99%) in both the GWAS and the replication set.

**Figure 2:**
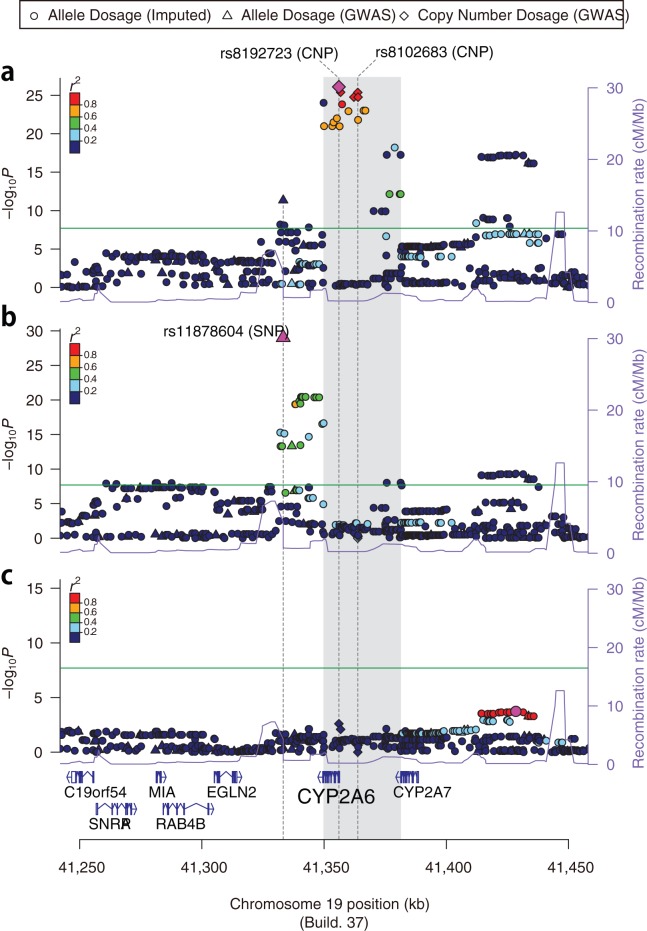
Signal plots before conditioning (a), after conditioning on rs8102683 (b) and after conditioning on rs8102683 and rs11878604 (c). SNPs and CNPs are plotted on the 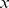 axis according to their positions on chromosome 19 (NCBI Build 37; hg19) against association with CPD on the left 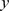 axis (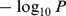-value). SNPs from the 1000 genomes imputation (phase I; 2011-11-23) are indicated with circles, SNPs genotyped on the Illumina 610K chip are indicated with triangles and CNPs genotyped with PlatinumCNV using the raw signal intensity data from the chip are indicated with diamonds. SNPs in the commonly deleted region (shown by the gray shaded area) are imputed as tri-allelic SNPs with deletions (see Materials and Methods for details). All SNP associations are assessed using allele dosages (the number difference between the A and B alleles; see Materials and Methods), and the CNP associations are assessed using the posterior mean copy number dosage [8]. Recombination rates (cM/Mb) across the region are shown by the purple line plotted against the right 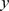 axis. The most significant variant of the SNP or CNP for each panel is pink, and the surrounding SNPs and CNPs are color-coded to reflect the strength of LD with the top variant according to the Pearson's 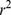 values.

Because multiple SNPs other than the CNPs at the *CYP2A6* locus also showed significant association with CPD (Figure S4), we performed fine-scale imputation analysis for our GWAS set using the 89 Japanese subjects of the 1000 Genomes Project as a reference (Phase I 2011-11-23). We selected SNPs within a 1 cM region around the CNP (rs8102683), which encompasses a series of CYP2 family genes (Figure S4). Here, the common deletion at the *CYP2A6* locus was addressed properly by the imputation study; we assumed that each SNP within the commonly deleted region consisted of two normal SNP alleles along with the common deletion allele. Therefore, the imputation treated the SNPs as tri-allelic markers (see Materials and Methods for details). As a consequence, none of the imputed SNPs showed greater significance than the CNP in the GWAS (Figure 2a), suggesting there are no causal SNPs in absolute LD (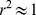) with the CNP.

To investigate whether the association between CPD and the *CYP2A6* locus can be explained completely by the observed CNP, we carried out tests of association for all CNPs and SNPs spanning the *CYP2A6* locus conditional upon this CNP (Figure 2b). Surprisingly, we found that a SNP (rs11878604; 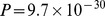) 30 kb downstream of the CYP2A6 gene was a secondary marker, which explains the additional effect of *CYP2A6* locus on CPD. Further conditioning on rs11878604 after conditioning on the CNP (rs8102683) did not show any obvious signal of association in the region (Figure 2c), suggesting that the major effect of the *CYP2A6* locus on CPD can be explained by the combination of the CNP and rs11878604.

To better understand the genetic architecture of the *CYP2A6* locus, we assessed the CPD association in terms of haplotypes underlying the CNP (rs8102683) and rs11878604 under the assumption of additive effect (Figure S7). Estimated haplotype frequencies revealed that the deletion (0 copies) at the CNP and the mutant allele rs11878604[C] are almost mutually exclusive (Table 1). Haplotypes including either of these variants showed a similar effect on CPD when compared with the reference haplotype (one copy at the CNP and the ancestral allele rs11878604[T]). Hence, individuals with a haplotype of the deletion (0 copies) and the ancestral allele rs11878604[T] (we refer to this as the “deletion haplotype”) require an average of four fewer cigarettes to maintain the same nicotine level as individuals who are homozygous for the reference haplotypes (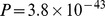). Likewise, individuals with a haplotype of the mutant allele rs11878604[C] and one copy at the CNP (we refer to this as the “mutant haplotype”) also require an average of three fewer cigarettes to maintain the same nicotine level as individuals who are homozygous for the reference haplotypes (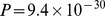). These patterns were completely replicated in the replication set (Table 1).

**Table 1:** Frequency and relative effect size of haplotype between rs11878604 (SNP) and rs8102683 (CNP).

Haplotype¶		GWAS set			Replication set		
rs11878604 (SNP)	rs8102683 (CNP)	Haplotype Freq.* (%)	Relative effect size* (s.e.)	*P* *	Haplotype Freq.* (%)	Relative effect size* (s.e.)	*P* *
T	1 copy	41.41	*reference*		41.8	*reference*	
T	0 copy	19.22	−4.00 (0.290)	3.8×10^−43^	18.6	−4.30(0.384)	8.3×10^−29^
C	1 copy	38.14	−2.69 (0.237)	9.4×10^−30^	38.4	−2.63(0.309)	2.3×10^−17^
C	0 copy	0.76	−5.05 (1.548)	1.1×10^−3^	0.63	−3.84(2.200)	0.081
C	2 copies	0.23	−3.07 (2.900)	0.30	0.24	−2.62(3.612)	0.47
T	2 copies	0.28	−0.29 (2.413)	0.90	0.31	−0.414(2.879)	0.89
Explained variance†		1.83% (ANOVA F-test *P* = 9.5×10^−52^)			2.23% (ANOVA F-test *P* = 2.8×10^−30^)		

¶ Haplotypes consisting of alleles at rs11878604 [T/C] and haplotypic copy numbers at rs8102683 [0/1/2].

* Haplotype frequencies and relative effect sizes are jointly evaluated using a haplotype-specific linear regression model as described previously [27]. All model parameters were estimated using a standard EM (expectation-maximization) algorithm [28].

† Variance explained by these six haplotypes and its significance was assessed in the standard ANOVA (analysis of variance) framework.

It has previously been suggested that the deletion of the CYP2A6 gene strongly down-regulates nicotine metabolism [12], and it is very common only in the Japanese population [14]. This allele is often referred to as CYP2A6*4, a classical *CYP2A6* functional allele (Human Cytochrome P450 Allele Nomenclature Committee; see URLs), which has been captured by the CNP (rs8102683) in our study. However, the secondary SNP (rs11878604) located in an intergenic region has been unknown a priori in any previous study and may not have any direct functional impact on the CYP2A6 gene. Therefore, we investigated the LD between rs11878604 and the *CYP2A6* functional alleles using phased haplotype data from the imputation reference panel (Table S5), which included several known deleterious SNPs in the CYP2A6 gene. Consequently, we found that the mutant haplotype is linked to the functional alleles CYP2A6*7 (or possibly *36 or *37), *9 (or possibly *15) and *10, all of which are known to strongly down-regulate nicotine metabolism [12]. This observation is not surprising because the mutant haplotype strongly reduced daily cigarette consumption (Table 1).

It was previously shown that the *CYP2A6* alleles are associated with smoking-related lung cancer in a Japanese population [12]. Therefore, we also assessed the association between the *CYP2A6* locus and chronic obstructive pulmonary disease (COPD; 982 cases and 4,480 controls), lung cancer (997 cases and 6,491 controls) and arteriosclerosis obliterans (ASO; 499 cases and 10,975 controls) in our GWAS and replication sample sets. Compared with the reference haplotype, we observed significant associations of the deletion haplotype with COPD (OR  = 0.2, 95%CI 0.14–0.27, 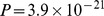) and lung cancer (OR  = 0.33, 95%CI 0.25–0.45, 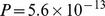), as well as a suggestive association with ASO (OR  = 0.53, 95%CI 0.36–0.77, 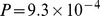) (Table 2). Similarly, we observed significant associations of the mutant haplotype with COPD (OR  = 0.38, 95%CI 0.3–0.49, 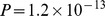) and lung cancer (OR  = 0.55, 95%CI 0.44–0.69, 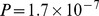) (Table 2).

**Table 2:** Association of the haplotype between rs11878604 (SNP) and rs8102683 (CNP) for three dieseases.

Haplotype¶		COPD (982 cases, 4,480 controls)					Lung Cancer (997 cases, 6,491 controls)				ASO (499 cases, 10,975 controls)			
rs11878604 (SNP)	rs8102683 (CNP)	case (%)	ctrl (%)	OR^*^ (95%CI)		*P* ^*^	case (%)	ctrl (%)	OR^*^ (95%CI)	*P* ^*^	case (%)	ctrl (%)	OR^*^ (95%CI)	*P* ^*^
T	1 copy	51.65	39.61	*reference*			50.30	41.15	*reference*		45.69	41.11	*reference*	
T	0 copy	13.49	19.76	0.20(0.14–0.27)	3.9×10^−21^		14.24	19.47	0.33(0.25–0.45)	5.6×10^−13^	16.29	19.41	0.53(0.36–0.77)	9.3×10^−4^
C	1 copy	33.95	39.45	0.38(0.30–0.49)	1.2×10^−13^		34.53	38.12	0.55(0.44–0.69)	1.7×10^−7^	36.71	38.19	0.72(0.54–0.97)	0.028
C	0 copy	0.36	0.64	0.40(0.04–3.85)	0.43		0.44	0.76	0.29(0.04–2.03)	0.21	0.73	0.77	0.99(0.15–6.77)	0.99
C	2 copies	0.21	0.23	3.28(0.11–95.47)	0.49		0.23	0.25	1.25(0.06–27.88)	0.89	0.24	0.23	0.77(0.02–31.39)	0.89
T	2 copies	0.34	0.31	0.53(0.05–5.88)	0.60		0.26	0.27	0.24(0.02–3.33)	0.29	0.34	0.29	1.24(0.10–16.04)	0.87
Liability variance^†^		4.0% (Likelihood Ratio–test *P* = 1.6×10^−23^)					0.71% (Likelihood Ratio–test *P* = 3.8×10^−13^)				0.23% (Likelihood Ratio–test *P* = 0.026)			

Because the relative effects of the haplotypes were very large, we further assessed the explained phenotypic variance of CPD (Table 1). We observed that the haplotypes at the *CYP2A6* locus significantly explain 2% of the phenotypic variance in CPD (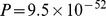 for the GWAS set). We also found that the liability variance explained by the haplotypes to be 4% and 1% for COPD and lung cancer, respectively (likelihood ratio test 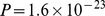 and 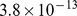, respectively).

## Discussion

The observations in this study highlighted several important issues regarding the missing heritability [15] that have not yet been explained by current GWAS. Despite the fact that the 1000 Genomes Project covered almost all SNPs in the human genome, the high frequency deletion (CYP2A6*4) confirmed in our study was not tagged by nearby SNPs (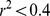). This finding suggests that the high similarity between sequences around *CYP2A6* may have caused recurrent deletion events in multiple ancestors in the Japanese population (see reference [16] for a detailed explanation of the mechanism underlying recurrent deletion events). Although recent studies have claimed that common CNPs may be indirectly explored through genome-wide SNP studies [17], [18], we believe that some common CNPs might have been missed by the current genome-wide SNP studies, and those CNPs may still account for a percentage of the missing heritability.

Another important aspect of this study is that the tag-SNP (rs11878604) is located in an intergenic region, which resulted in the collapse of the functional effect of multiple deleterious SNPs (two nonsense SNPs in exon 9 and a SNP in TATA box of *CYP2A6*) that had the most significant associations with CPD after conditioning on the CNP (rs8102683). In these cases, we would generally employ a fine-mapping approach to investigate the region using imputation with data from the 1000 Genomes project or deep resequencing of nearby genes. However, if the tag-SNP was associated with a number of rare variants in a gene or a gene cluster, the single SNP test after imputation or resequencing may not be able to fully explain the underlying phenomena because the common tag-SNP will still be the most powerful marker in the region. Therefore, we may have to take into account the effect of multiple deleterious SNPs for regions in which haplotype analysis and collapsing methods [19]–[22] play an important role.

We also assessed the association of previously reported loci using our GWAS data (Table S6). Interestingly, while previous studies indicated that the region with the strongest association with CPD was the 15q25 cluster, which encompasses the CHRNA3, CHRNA5 and CHRNB4 genes [4]–[6], our study revealed that the *CYP2A6* locus has the strongest association with CPD in the Japanese population. The 15q25 cluster includes genes coding for nicotinic acetylcholine receptors; however, the Japanese population seems to have less genetic variation in this area than the European population ( *i.e*., a lower minor allele frequency at the CPD associated locus; Table S6). It is also interesting that the common deletion at the *CYP2A6* locus was significant in the Japanese population but not in the European population [14]. These findings suggest that there might have been a strong selective pressure [23] on the 15q25 locus in the Japanese population and on the *CYP2A6* locus in the European population, although no evidence for this selection has been observed using the HGDP selection browser (see URLs).

In summary, our study identified a significant association between CNP-SNP haplotypes at the *CYP2A6* locus and CPD in a Japanese population. Although this locus has been investigated extensively using the candidate gene approach ( *e.g*., [12]), our study adds to the understanding of the genetic basis of smoking behavior and smoking-related diseases in the Japanese population.

## Materials and Methods

### Ethics Statement

The subjects who participated in the GWAS and the replication study were enrolled by the BioBank Japan Project [7] at the Institute of Medical Science, the University of Tokyo. All participants provided written informed consent as approved by the ethical committees of the RIKEN Yokohama Institute and the Institute of Medical Science, the University of Tokyo. This study has been approved by the ethical committees of the RIKEN Yokohama Institute and the Institute of Medical Science, the University of Tokyo.

### Samples

The subjects who participated in the GWAS (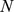 = 11,696) and the replication study (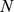 = 5,462) were enrolled by the BioBank Japan Project [7] (Table S1). Subjects who were determined to be of non-Japanese origin by self-report or by PCA and IBS (identity-by-state) analysis were not included. Clinical information, including subject age, gender and smoking history (smoking quantity, age of smoking cessation and age of smoking initiation), was collected using a standard questionnaire (Table S1).

In this study, we have selected subjects as smokers if they had ever smoked, as reported on the smoking initiation history. We used both former and current smokers in the GWAS and the replication study according to smoking cessation history, with a proper treatment in the linear regression model (see statistical methods below). We excluded subjects with invalid CPD information ( *e.g*., 5–10 cigarettes/day or 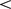
5 cigarettes/day). We also exclude subjects below the age of twenty or who did not provide their sex.

### SNP Genotyping and Quality Control

SNP genotyping was performed using the Illumina HumanHap610-Quad Genotyping BeadChip (Illumina, CA, USA) for the GWAS, which was conducted for 19 diseases (Table S1). After excluding subjects with call rates lower than 0.98, we excluded SNPs with call rates lower than 0.99, SNPs with ambiguous calls, and non-autosomal SNPs. We also excluded closely related subjects using IBS. For each pair with 1st or 2nd degree kinship, we excluded the individual with lower call rates. We also excluded subjects whose ancestries were estimated to be distinct from the other subjects using PCA performed by EIGENSTRAT (version 2.0). We performed PCA on the SNP genotype data from our study along with the genotype data from unrelated European (CEU), African (YRI), and East-Asian (Japanese and Han Chinese; JPT + CHB) individuals obtained from the Phase II HapMap database (release 24). Based on the PCA plot, we excluded subjects who fell outside the JPT and CHB clusters (Figure S1). We then excluded SNPs with a MAF of 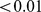 or SNPs with Hardy-Weinberg equilibrium (HWE) test P-value 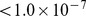 (Fisher's exact test). Ultimately, we obtained genotype data on 480,103 SNPs for 11,696 subjects who had also completed the questionnaire. Genotype imputation was then performed using a two-step procedure in MACH 1.0. The JPT and CHB data obtained from the Phase II HapMap database (release 24) were used as references. In the first step, crossover and error rate maps were estimated using 500 subjects who were randomly selected from the GWAS data. In the second step, genotype imputation of all subjects was conducted using the crossover and error rate maps estimated in the first step. We excluded imputed SNPs with MAFs of 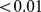 or 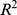 values of 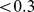 and obtained SNP genotype data for 2,312,503 SNPs.

We used genotyping data collected using the Illumina OmniExpress II Genotyping BeadChip (Illumina, CA, USA) for the replication study set, which covered 16 diseases (Table S1). We applied the same quality control criteria and imputation procedure as for the GWAS data.

### CNP Genotyping and Quality Control

CNP genotyping for the GWAS and replication sets was performed using the PlatinumCNV [8], which uses a Bayesian Gaussian mixture model for SNP array signal intensity data. We ran PlatinumCNV on the raw signal intensity data from the Illumina 610K platform (GWAS set) and obtained 4,256 CNP loci that passed the following quality control criteria: call rate (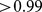), HWE test (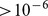) and minor allele frequency of total aneuploidy haplotypes (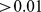) (calculated as described in [8]). For the replication set, we ran PlatinumCNV on the signal intensity data of markers within the *CYP2A6* locus. The five CNPs that satisfied the genome-wide significance level (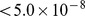) in the GWAS were successfully genotyped using the signal intensity data from the OmniExpress II platform (see Figure S8 for detailed clustering results).

To assess the accuracy of the result of PlatinumCNV, we randomly selected 445 and 759 subjects from the GWAS and replication sets, respectively. The copy number for each individual from the TaqMan Assay was compared with the maximum a posteriori (MAP) genotype (see [8] for details) obtained using the PlatinumCNV, and the concordance rate was calculated for each of the five CNP markers at the *CYP2A6* locus (Table S4).

### TaqMan Assays

We performed TaqMan gene copy number assays using quantitative PCR following a previously described method [24]. All primers and TaqMan probes were designed with Primer Express software v2.0 (Applied Biosystems, Tokyo, Japan). The commercially available RNase P assay (Applied Biosystems, Tokyo, Japan) was used as a copy number reference gene. All assays were performed on an ABI 7900HT (Applied Biosystems, Tokyo, Japan) using TaqMan Gene Expression PCR Master Mix (Applied Biosystems, Tokyo, Japan) with 5 ng of genomic DNA in a 10 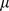
l reaction. The cycling conditions were the following: 95 degrees Celsius for 10 min for the initial denaturation and enzyme activation, followed by 40 cycles of 95 degrees Celsius for 15 sec and 60 degrees Celsius for 1 min. The copy number was calculated using the comparative *Ct* method [25], in which *Ct* indicates the threshold cycle.

### Association Studies

In the GWAS and the replication study, the association of each CNP or SNP with CPD was assessed using a linear regression model that assumed additive effects for copy number dosage or allele dosage (ranging from 0.0 to 4.0 for CNPs and from 0.0 to 2.0 for SNPs, respectively). In the regression model, gender, age, age-squared, smoking cessation status (former/current), status for the 19 diseases and the first and second principal components (PCs) in the East Asian population (Figure S2; see reference [26] for details of the Japanese population structure) were adopted as covariates. In the replication study, the most significantly associated CNPs and SNPs in the loci that had 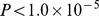 in the GWAS were evaluated using R statistical software. For the CNP and the SNPs in the combined GWAS and replication study, associations were evaluated using the results of the genome-wide meta-analysis for CPD. The combined results of the studies were obtained using an inverse-variance method from the summary statistics 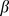 (effect size) and the standard error.

### Haplotype Analysis

We also performed haplotype analyses for CPD and the smoking-related diseases (COPD, lung cancer and ASO) using the GWAS and the replication samples. We assumed six haplotypes at the *CYP2A6* locus, each of which consisted of a combination of an allele at rs11878604[T/C] and a haplotypic copy number at rs8102683[0/1/2]. Here, the MAP copy number dosage [8] was used as the copy number genotype. Population haplotype frequencies and their relative effect on CPD were jointly evaluated using the haplotype-specific linear regression model, as described previously [27]. In this model, we used the same covariates that were used in the GWAS and the replication study. All model parameters, including the effect sizes of the six haplotypes, were estimated using a standard EM (expectation-maximization) algorithm [28].

For the disease traits, the odds ratios of the haplotypes were evaluated by the haplotype-specific logistic regression model [27], and the amount of liability variance explained by the haplotypes was calculated using the standard liability threshold model [29]. The statistical significance of the variance was assessed using a likelihood ratio (LR) test between the null model and the full model with the haplotypes; the covariates of gender, age, age-squared, smoking cessation status (former/current) and the first and second PCs for the East Asian population were used in both of the models.

### Inference of Common Deletion for Reference Sample

We used the 89 Japanese subjects from the 1000 Genomes Project as a reference, because the deletion frequency at the *CYP2A6* locus may vary between different ethnic groups [14]. The common deletion polymorphism for the reference sample was inferred, and the breakpoints were estimated for the commonly deleted region. We assessed the “depth of coverage” for each subject, which was obtained from the alignment read data available from the 1000 Genomes Project. The depth of coverage for each subject was extracted using GATK software [30] and normalized to the mean coverage on chromosome 19 (Figure S6). The normalized depth in the 41.35–41.38 Mb region on chromosome 19 was used to calculate singular values that reflect the absolute numbers of copies for the individuals at the region (Figure S9). Then, a standard Gaussian mixture model was fitted to the singular values, and the copy number was inferred for each individual (a similar method was established previously [31]). The accuracy of the results was evaluated using the TaqMan assay, and the concordance rate for the 45 (out of 89) subjects tested was 100% (Figure S9).

### Breakpoint Estimation for Common Deletion

The breakpoints of the commonly deleted region were estimated using a hidden Markov model. We calculated the Pearson's correlation coefficient 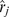 between the inferred copy number genotype and normalized depth at base 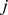. If the base 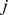 belongs to the commonly deleted region, then the true underlying correlation coefficient should be 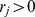; otherwise, it should be 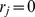. It is apparent that the squared correlation coefficient multiplied by the sample size (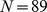) asymptotically follows a 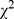 distribution with a non-central parameter 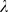 and one degree of freedom. Therefore, we fitted a hidden Markov model with the following two states: one corresponds to the null hypothesis of the test of Pearson's correlation (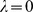), and the other corresponds to the alternative hypothesis (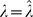). We used a standard Baum-Welch algorithm [32] to estimate 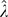 and determined the maximum likelihood breakpoints located at 41,349,714 (95% CI: 41,349,709–41,349,715) and 41,381,486 (95% CI: 41,381,478–41,381,488) (Figure S10).

### Imputation and Association Study at the *CYP2A6* Locus

We used genotype data of the 89 Japanese subjects from the 1000 Genomes Project (phase I; 2011-11-23) as a reference panel for the imputation study to perform fine mapping of the *CYP2A6* locus in relation to CPD. We selected SNPs within a region of 1 cM across the most significant CNP (Figure S4). For SNPs within the commonly deleted region, the reference genotype data were updated to reflect the inferred copy number (Table S7). For individuals with zero copies of the CYP2A6 gene, genotypes within the region were represented as homozygote pairs of the deletion allele (referred to as “O”). For individuals with one copy of the CYP2A6 gene, genotypes were represented as heterozygous pairs of a normal SNP allele (referred to as “A” or “B”) and the deletion allele. Heterozygous genotypes of normal SNP alleles were represented as having an unknown genotype because we were unable to infer which of the two normal alleles were inherited by each individual (these unknown genotypes in the reference panel were also imputed during the imputation).

We performed imputation using the reference genotype in combination with the CNPs and the SNPs in the GWAS. We selected markers within a 1 cM region around the top CNP (Figure S4). The SNPs in the commonly deleted region were then imputed as if they were tri-allelic markers (Figure S11). The Beagle software [33] used played an important role because it can perform multi-allelic imputation. The association of these tri-allelic SNPs with CPD was evaluated using a linear regression model in conjunction with the covariates used in the GWAS. Here, the allele dosage of the difference in the number of A and B alleles (ranging from 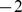 to 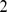) was assessed with the assumption of an additive effect on CPD, as proposed previously [34].

To assess LD between rs11878604 and the classical functional alleles of the CYP2A6 gene, we simply extracted the seven missense SNPs in *CYP2A6* from the reference panel. We assigned the known functional alleles to the haplotypes according to the pattern of the mutant alleles on the home page of the human *CYP* allele nomenclature committee (see URLs) and calculated frequencies in conjunction with the alleles at rs11878604.

### URLs

The URLs for data presented herein are as follows:

The BioBank Japan Project, http://biobankjp.org;

EIGENSTRAT software, http://genepath.med.harvard.edu/reich/Software.htm;

MACH, http://www.sph.umich.edu/csg/abecasis/MACH/index.html;

International HapMap Project, http://www.hapmap.org;

R statistical software, http://cran.r-project.org;

Human Cytochrome P450 (*CYP*) Allele Nomenclature Committee, http://www.cypalleles.ki.se/;

PlatinumCNV, http://kumasakanatsuhiko.jp/projects/platinumcnv/;

UGDP selection growser, http://hgdp.uchicago.edu/cgi-bin/gbrowser/HGDP/;

1000 Genomes Project, http://www.1000genomes.org/.

## Supporting Information

Figure S1 **Distribution of the subjects in the results of PCA analysis.** Two dimensional display of the first two eigenvectors of the subjects finally enrolled in the genome-wide association study (GWAS) for CPD (n = 17,351), and HapMap European (CEU), African (YRI), Han Chinese (CHB) and Japanese (JPT) samples (Phase II, release 24; [35]) in the results of principal component analysis (PCA [36]). The eigenvectors clearly separated the subjects into three clusters (YRI, CEU, and JPT + CHB clusters), and the distribution of the subjects in the GWAS was concordant with the JPT + CHB cluster as previously anticipated in the Japanese population [37].
(EPS)

Figure S2 **Distribution of the subjects in the results of PCA analysis within East-Asia.** Two dimensional display of the first two eigenvectors of the subjects finally enrolled in the genome-wide association study (GWAS) for CPD (n = 17,351), HapMap Han Chinese (CHB) and Japanese (JPT) samples (Phase II, release 24; [35]) in the results of principal component analysis (PCA [36]). The eigenvectors clearly separated the subjects into three clusters (Hondo, Ryukyu, and CHB clusters; [37]). These two vectors were used as covariates in the linear regression model of CPD.
(EPS)

Figure S3 **Quantile-Quantile plots of P-values in the GWAS of CPD with genome-wide SNPs (a) and CNPs (b).** The horizontal axis indicates the expected −log10(P-values) and the vertical axis indicates the observed −log10(P-values). The diagonal line represents y = x, which corresponds to the null hypothesis, and the region colored in blue shows 95% confidence interval based on Beta distribution [38].
(EPS)

Figure S4 **Main region of associateion on 19q13.** The upper part of plot shows the −log10P value of the association for CPD in the GWAS. The P values were caluculated using CNPs genotyped by PlatinumCNV (red triangles), SNPs on Illumina 610K chip (yellow lower triangles) and SNPs imputed using HapMap reference panel (blue circles). The lower panel shows the fine-scale recombination rate across the region (the right y-axis; purple) and cumulative recombination rate (the left y-axis; yellow) measured away from the representative marker (rs8102683) of the most highly associated CNPs whose location is indicated by the dotted line at the middle of the plot. The vertical broken lines on the plot indicate the main region of association whose genetic distance is approxmately 1 cM across the marker CNP. We used SNPs on the Illumina 610K platform within the region for the subsequent 1000 Genomes imputation (see Materials and Methods for details).
(EPS)

Figure S5 **Linkage disequilibrium plot of five CNPs around CYP2A6 gene.** The upper triangle shows Pearson's correlation coefficients of the postrior mean copy number doasages among the five CNPs for the Illumina OmniExpress II platform, and the lower triangel shows those for the Illumina 610K platform.
(EPS)

Figure S6 **Depth of coverage in the Japanes sample of the 1000 Genomes Project (phase I).** The depth of coverage for each subject (JPT; N = 89) was obtained using the GATK software [30]. Coverage is normalized by the mean coverage for each subject on chromosome 19 and then multiplied by 2 so that the normilized coverage is concentrated around the normal copy number of two (y-axis). The curve represents a fitted local regression smoother to the normalized depth for each subject using LOESS (window size  = 2 Kbp). The depth of coverage drops between 41.35–41.38 Mb correspond with known deletion polymorphism in Japanese [12]. Line color indicates the number of copies at the known deletion polymorphism determined by a Gaussian mixture model given in Supplementary Figure 9. The dashed lines indicate chromosomal positions for the five CNP markers in the Illumina 610K chip.
(EPS)

Figure S7 **Avarage CPD against the number of copies at rs8102683 and SNP genotype at rs11878604.** The y-axis shows the CPD adjusted by the covariates (age, age-square, gender, affection status, smoking cessation status, first and second principal components). The plot clearly shows that the larger the copy number is the more the CPD is. The plot also suggests the mutant allele rs11878604[C] strongly down-regulates nicotine metabolism. Overall, the deletion or the mutant allele rs11878604[C] acts in an additive fashion to CPD.
(EPS)

Figure S8 **Fluorescent signal intensity plots for five CNPs.** The left column indicates signal intensities observed from the Illumina 610K platform and the right column indicaes those from the Omni Express II. The fitted Gaussian mixture model is shown by ellipses with colors indicating the different copy number genotypes (blue: 0 copy; green: 1 copy; gray: 2 copies; orange: 3 copies; pink 4 copies).
(EPS)

Figure S9 **Histgram of singular values calculated from the normalized depth of coverage.** The histgram shows a result of the singular value decomposition of the normalized depth for the 89 Japneses between 41.35–41.38 Mb region. A Gaussian mixture model is then fitted on the singular value to infer the copy number for each subject (the similar method is established in [31]). The inferred copy numbers for 45 out of 89 subjects are validated by qPCR (filled circles below the histogram), and the concordance rate is 100%.
(EPS)

Figure S10
**Common breakpoint estimation using a hidden Markov model.** We first calculate the Pearson's correlation coefficient *r_j_* between the normalized depth at base *j* and the inferred copy number genotype (Supplementary Figure 9). Then the squred correlation coefficient multiplied by the sample size of n = 89, that is 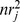 (y-axis), asymptotically follows a *x* ^2^ distribution with a noncentral parameter λ and one degree of freedom. The non-central parameter λ at base *j* is essentially determined whether the base *j* belongs to the CNV region (*r_j_*>0) or not (*r_j_* = 0). We thus fit a hidden Markov model with two hidden states, one corresponds to the null hypothesis for the test of Peason's correlation with λ = 0, and the other is the alternative hypothesis with 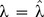. We use a standard Baum-Welch algorithm [32] with transition probabilities *p*(*Null*|*Alt*.)  =  *p*(*Alt.*|*Null*)  = 10^−250^ to estimate 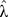 and {*p*(*Alt.*), *p*(*Null*)}. We then obtain the maximum likelihood path to estimate common breakpoints of the copy number polymorphism. The blue bars indicates the maximum likelihood path correspond with the null hypothesis at the mean of null distribution (*y* = 1). The red bar indicates that with the alternative hypothesis at the mean of the althernative distribution (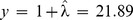). The common breakpoints are located at 41,349,714 (95%CI: 41,349,709–41,349,715) and 41,381,486 (95%CI: 41,381,478–41,381,488), respectively.(EPS)

Figure S11 **Stylized picture of imputation within a common deletion region.** We assume there exist two normal SNP alleles “A” and “B” in conjunction with the deletion allele “O”. Normally, the imputation method is applied to biallelic markers in which a hidden haplotype state consists of a combination of the two normal alleles (top panel). Once imputed SNPs were existing in a common deletion region (bottom panel), the haplotype within this region may switch from the two normal alleles to the deletion allele.
(EPS)

Table S1 **Characteristics and distributions of the traits in the study population.**
(PDF)

Table S2 **Replication study of significant and suggestive genetic loci in GWAS.**
(PDF)

Table S3 **Haplotype frequency for five CNP markers.**
(PDF)

Table S4 **Results of TaqMan validation for GWAS and replication set.**
(PDF)

Table S5 **Haplotype frequencies of CYP2A6 functional allele and rs11878604.**
(PDF)

Table S6 **Results of the GWAS and the replication study for loci previously studied with European populations.**
(PDF)

Table S7 **Reference genotype replacement rule in a common deletion region.**
(PDF)
