# Supplementary material for: Haplotypes with Copy Number and Single Nucleotide Polymorphisms in CYP2A6 Locus Are Associated with Smoking Quantity in a Japanese Population
Source: PLoS One. 2012 Sep 25;7(9):e44507. doi: 10.1371/journal.pone.0044507 (PMC3458030; doi:10.1371/journal.pone.0044507)
Supplement: Table S1 — Characteristics and distributions of the traits in the study population. (PDF) [file pone.0044507.s012.pdf]

Table S1. Characteristics and distributions of the traits in the study population.

Stage	Genotyping platform	No. subjects	Age (mean ± SD)	No. female (%)	CPD (mean ± SD)
GWAS set*	Illumina HumanHap 610K	11,696	62.8 ± 11.1	2,296 (19.7)	24.6 ± 16.8
Replication set†	Illumina OmniExpress II	5,462	55.3 ± 15.1	1,390 (25.3)	23.2 ± 15.3

* Subjects consisted of Japanese patients in 19 disease groups (not mutually exclusive) as follows: Colorectal cancer (*N*=1286), Breast cancer (*N*=335), Prostate cancer (*N*=869), Lung cancer (*N*=997), Gastric cancer (*N*=1,167), Diabetes (*N*=3,169), Atherosclerosis obliterans (ASO) (*N*=721), Arrhythmia (*N*=1,265), Cerebral infarction (*N*=1,180), Myocardial infarction (*N*=1,651), Gallbladder and Bile duct carcinoma (*N*=55), Pancreas cancer (*N*=107), Drug eruption (Drug-induced hypersensitivity syndrome) (*N*=38), Rheumatoid arthritis (*N*=590), Amyotrophic lateral sclerosis (ALS) (*N*=4), Liver cancer (Hepatocellular carcinoma) (*N*=705), Liver cirrhosis (*N*=952), Osteoporosis (*N*=211), Uterine myoma (Myoma uteri) (*N*=409)

† Subjects consisted of Japanese patients in 16 disease groups (not mutually exclusive) as follows: Diabetes (*N*=511), Bronchial asthma (*N*=241), Basedow's disease (*N*=800), Cerebral infarction (*N*=246), Cerebral aneurysm (*N*=599), Emphysema (Chronic obstructive pulmonary disease; COPD) (*N*=982), Atopic dermatitis (*N*=443), Hyperlipidemia (*N*=583), Arrhythmia (*N*=237), Cataract (*N*=329), Urolithiasis (*N*=650), Glaucoma (*N*=363), Epilepsy (*N*=653), Esophageal cancer (*N*=705), Uterine body cancer(*N*=125), Nephrotic syndrome (*N*=320)
