# Supplementary material for: Haplotypes with Copy Number and Single Nucleotide Polymorphisms in CYP2A6 Locus Are Associated with Smoking Quantity in a Japanese Population
Source: PLoS One. 2012 Sep 25;7(9):e44507. doi: 10.1371/journal.pone.0044507 (PMC3458030; doi:10.1371/journal.pone.0044507)
Supplement: Table S2 — Replication study of significant and suggestive genetic loci in GWAS. (PDF) [file pone.0044507.s013.pdf]

Table S2. Replication study of significant and suggestive genetic loci in GWAS.

		Position	Coded	GWAS set		Replication set		Combined	
SNP	Chr	(hg19)	allele	Effect (s.e.)	*P*	Effect (s.e.)	*P*	Effect (s.e.)	*P*
rs8102683	19	41,363,765	Copy Number	2.68 (0.253)	4.3×10^{-26}	2.89 (0.338)	1.5×10^{-17}	2.76 (0.203)	3.8×10^{-42}
rs2106595	7	117,946,785	G	1.2 (0.24)	9.5×10^{-7}	0.35 (0.32)	0.28	0.88 (0.19)	4.9×10^{-6}
rs12317231	12	1,580,082	G	-2.7 (0.61)	9.3×10^{-6}	0.3 (0.73)	0.68	-1.5 (0.47)	0.0016
rs12438196	15	49,450,054	T	-1.5 (0.32)	3.9×10^{-6}	0.21 (0.45)	0.65	-0.91 (0.26)	5.0×10^{-4}
rs4812020	20	56,597,486	G	-1.1 (0.24)	6.5×10^{-6}	-0.12 (0.28)	0.66	-0.68 (0.18)	0.00021
rs4925446	22	47,485,894	T	1.2 (0.26)	8.1×10^{-6}	0.14 (0.34)	0.69	0.79 (0.21)	0.00015
