# Supplementary material for: Haplotypes with Copy Number and Single Nucleotide Polymorphisms in CYP2A6 Locus Are Associated with Smoking Quantity in a Japanese Population
Source: PLoS One. 2012 Sep 25;7(9):e44507. doi: 10.1371/journal.pone.0044507 (PMC3458030; doi:10.1371/journal.pone.0044507)
Supplement: Table S3 — Haplotype frequency for five CNP markers. (PDF) [file pone.0044507.s014.pdf]

Table S3. Haplotype frequency for five CNP markers.

Haplotype					Freq. (%)
rs8192723	rs10418304	rs10422346	rs8102683	rs8105704	
1	1	1	1	1	78.0
0	0	0	0	0	18.0
1	1	0	1	1	0.56
1	0	0	0	0	0.56
0	1	1	1	1	0.51
0	1	0	0	0	0.37
1	1	2	1	1	0.33
1	0	1	1	1	0.29
0	0	1	0	0	0.24
1	1	1	2	1	0.23
1	1	0	0	0	0.17
0	0	0	0	1	0.15
1	1	1	0	1	0.15
1	1	1	2	2	0.13
1	1	1	1	0	0.091
1	1	1	1	2	0.079
1	1	2	2	2	0.056
1	1	2	2	1	0.026
0	1	2	1	1	0.024
1	0	1	0	0	0.021
0	1	0	1	1	0.019
0	1	0	0	1	0.017
1	0	0	0	1	0.013
1	0	1	1	2	0.0086
1	1	2	1	2	0.0082
1	1	0	1	2	0.008
0	1	1	0	0	0.0078
1	0	1	2	1	0.0077
1	1	0	0	1	0.0075
0	0	1	1	1	0.0049
1	1	2	1	0	0.0039
1	0	1	1	0	0.0039
1	1	1	0	0	0.0038
1	0	0	1	0	0.0035
0	1	1	0	1	0.0033
1	1	0	2	1	0.0027
1	0	0	1	1	0.002
1	2	0	2	2	1.0×10^{-23}
1	0	1	0	2	5.5×10^{-50}

Haplotype frequencies among the five CNPs (rs8192723, rs10418304, rs10422346, rs8102683, rs8105704) were estimated by a standard EM algorithm with the maxmum a posteriori copy number dosages obtained by PlatinumCNV.
