# Supplementary material for: Haplotypes with Copy Number and Single Nucleotide Polymorphisms in CYP2A6 Locus Are Associated with Smoking Quantity in a Japanese Population
Source: PLoS One. 2012 Sep 25;7(9):e44507. doi: 10.1371/journal.pone.0044507 (PMC3458030; doi:10.1371/journal.pone.0044507)
Supplement: Table S4 — Results of TaqMan validation for GWAS and replication set. (PDF) [file pone.0044507.s015.pdf]

Table S4. Results of TaqMan validation for GWAS and replication set.

	Concordance rate at each CNP locus (%)				
	rs8192723 (41,356,054*)	rs10418304 (41,356,751*)	rs10422346 (41,362,173*)	rs8102683 (41,363,765*)	rs8105704 (41,363,898*)
GWAS set† (*N*=445)	96.6	98.9	98.2	99.6	99.3
Replication set‡ (*N*=759)	96.7	97.8	97.4	99.2	98.8

We calculated the concordance rates by comparing the result of PlatinumCNV (maximum a posteriori copy number dosages) and the TaqMan validation result.

* NCBI Build. 37; hg19.

† qPCR assay position: 41,355,978-41,356,078 (Build 37; hg19)

‡ qPCR assay position: 41,363,935-41,364,006 (Build 37; hg19)
