# Supplementary material for: Haplotypes with Copy Number and Single Nucleotide Polymorphisms in CYP2A6 Locus Are Associated with Smoking Quantity in a Japanese Population
Source: PLoS One. 2012 Sep 25;7(9):e44507. doi: 10.1371/journal.pone.0044507 (PMC3458030; doi:10.1371/journal.pone.0044507)
Supplement: Table S5 — Haplotype frequencies of CYP2A6 functional allele and rs11878604. (PDF) [file pone.0044507.s016.pdf]

Table S5. Haplotype frequencies of *CYP2A6* functional allele and rs11878604.

Possible *CYP2A6* alleles†	rs11878604 T	rs11878604 C	Total(%)	P‡
*1, *2 and other functionl allels	35.96	3.37	39.33	*reference*
*4 (whole deletion)	19.10	2.25	21.35	0.74
*7, *36, *37 (missense SNP in Exon 9)	0.00	14.61	14.61	4.4×10^{-18}
*9, *15 (SNP in TATA box)	1.69	19.66	21.35	1.0×10^{-18}
*10 (two missense SNPs in Exon 9)	0.00	2.25	2.25	1.8×10^{-4}
*13	0.00	0.56	0.56	0.099
*18	0.00	0.56	0.56	0.099
Total(%)	56.75	43.25	100.0	

The phased reference panel data of 1000 Genomes project (phase I; 2011-11-23).

Only the Japanese subjects were used (JPT; $N = 89$).

† The *CYP2A6* functional alleles were determined by the seven nonsense SNPs of *CYP2A6* gene; rs28399468, rs5031017 (rs75344699), rs5031016 (rs60711313), rs61736436, rs1809810, rs28399434 and rs28399433. These SNP genotypes were phased during the imputation study using the Beagle software [33]. The known *CYP2A6* functional alleles (http://cypalleles.ki.se/) were assigned accoding to the combination of alleles in the phased haplotypes.

‡ Significance of association between each functional allele and the mutant allele at rs11878604 was assessed by the Fisher's exact test.
