# Supplementary material for: Haplotypes with Copy Number and Single Nucleotide Polymorphisms in CYP2A6 Locus Are Associated with Smoking Quantity in a Japanese Population
Source: PLoS One. 2012 Sep 25;7(9):e44507. doi: 10.1371/journal.pone.0044507 (PMC3458030; doi:10.1371/journal.pone.0044507)
Supplement: Table S6 — Results of the GWAS and the replication study for loci previously studied with European populations. (PDF) [file pone.0044507.s017.pdf]

Table S6. Results of the GWAS and the replication study for loci previously studied with European populations.

SNP	Chr	Position	Coded	GWAS set			Replication set			Combined			
		(hg19)	allele	Allele freq.	Effect (s.e.)	P	Allele freq.	Effect (s.e.)	P	Allele freq.	Effect (s.e.)	P	Ref.†
rs215605	7	32,336,965	T	0.31	−0.23 (0.22)	0.29	0.31	0.057 (0.29)	0.85	0.31	−0.13 (0.18)	0.47	[4]
rs215614	7	32,347,335	G	0.66	0.13 (0.22)	0.54	0.67	0.097 (0.29)	0.74	0.66	0.12 (0.17)	0.49	[4]
rs6474412	8	42,550,498	T	0.85	0.60 (0.30)	0.042	0.86	0.26 (0.4)	0.51	0.85	0.48 (0.24)	0.043	[4]
rs13280604	8	42,559,586	G	0.14	−0.56 (0.31)	0.073	0.13	−0.27 (0.4)	0.49	0.14	−0.45 (0.25)	0.066	[4]
rs1329650	10	93,348,120	T	0.76	0.07 (0.24)	0.77	0.76	−0.65 (0.32)	0.042	0.76	−0.20 (0.19)	0.31	[6]
rs1028936	10	93,349,797	C	0.62	−0.29 (0.22)	0.19	0.61	−0.18 (0.28)	0.51	0.61	−0.25 (0.17)	0.15	[6]
rs588765	15	78,865,425	T	0.12	−0.097 (0.32)	0.76	0.11	0.019 (0.42)	0.96	0.12	−0.055 (0.25)	0.83	[5]
rs16969968	15	78,882,925	G	0.98	−0.56 (0.71)	0.43	0.98	−1.50 (0.86)	0.088	0.98	−0.93 (0.55)	0.091	[5, 6]
rs1051730	15	78,894,339	G	0.98	−0.56 (0.71)	0.43	0.98	−1.50 (0.86)	0.088	0.98	−0.93 (0.55)	0.090	[4–6]
rs6495308	15	78,907,656	T	0.22	0.058 (0.25)	0.81	0.22	0.69 (0.32)	0.031	0.22	0.29 (0.20)	0.13	[5]
rs7937	19	41,302,706	T	0.64	0.19 (0.21)	0.37	0.64	0.13 (0.28)	0.65	0.64	0.17 (0.17)	0.32	[4]
rs3733829	19	41,310,571	G	0.39	0.72 (0.21)	0.00065	0.39	0.66 (0.28)	0.018	0.39	0.7 (0.17)	3.3×10^{-5}	[6]
rs1801272	19	41,354,533	T	0.012	−11 (2.40)	1.4×10^{-5}	0.012	−6.5 (3.70)	0.082	0.012	−9.4 (2.0)	4.5×10^{-6}	[4]
rs7260329	19	41,521,638	G	0.56	0.30 (0.21)	0.15	0.56	1.1 (0.27)	9.4×10^{-5}	0.56	0.59 (0.17)	0.00041	[4]
rs4105144	19	41,358,624	–	–	–	–	–	–	–	–	–	–	[4]

† previous reports provided in the main text.
