# Supplementary material for: Haplotypes with Copy Number and Single Nucleotide Polymorphisms in CYP2A6 Locus Are Associated with Smoking Quantity in a Japanese Population
Source: PLoS One. 2012 Sep 25;7(9):e44507. doi: 10.1371/journal.pone.0044507 (PMC3458030; doi:10.1371/journal.pone.0044507)
Supplement: Table S7 — Reference genotype replacement rule in a common deletion region. (PDF) [file pone.0044507.s018.pdf]

Table S7. Reference genotype replacement rule in a common deletion region.

Copy Number		Original data		
		AA	AB	BB
0	→	OO	OO	OO
1	→	AO	??	BO
2	→	BB	AB	BB

We assume there exist two normal alleles ″A″ and ″B″ along with the deletion allele ″O″ at the SNP locus within a copy number deletion region. Missing allele is indicated by ″?″.
